# Multi-omics analysis provides insights into lignocellulosic biomass degradation by *Laetiporus sulphureus* ATCC 52600

**DOI:** 10.1186/s13068-021-01945-7

**Published:** 2021-04-17

**Authors:** Fernanda Lopes de Figueiredo, Ana Carolina Piva de Oliveira, Cesar Rafael Fanchini Terrasan, Thiago Augusto Gonçalves, Jaqueline Aline Gerhardt, Geizecler Tomazetto, Gabriela Felix Persinoti, Marcelo Ventura Rubio, Jennifer Andrea Tamayo Peña, Michelle Fernandes Araújo, Maria Augusta de Carvalho Silvello, Telma Teixeira Franco, Sarita Cândida Rabelo, Rosana Goldbeck, Fabio Marcio Squina, André Damasio

**Affiliations:** 1 Department of Biochemistry and Tissue Biology, Institute of Biology, University of Campinas (UNICAMP), Campinas, SP Brazil; 2 Brazilian Biorenewables National Laboratory (LNBr), Brazilian Center for Research in Energy and Materials (CNPEM), Campinas, SP Brazil; 3 Department of Biological and Chemical Engineering (BCE), Aarhus University, 8200 Aarhus, Denmark; 4 Interdisciplinary Center of Energy Planning (NIPE), University of Campinas (UNICAMP), Campinas, SP Brazil; 5 Chemical Engineering School, University of Campinas (UNICAMP), Campinas, SP Brazil; 6 Department of Food Engineering, Faculty of Food Engineering, University of Campinas (UNICAMP), Campinas, SP Brazil; 7 Department of Bioprocess and Biotechnology, College of Agricultural Sciences, São Paulo State University (UNESP), Botucatu, SP Brazil; 8 Department of Technological and Environmental Processes, University of Sorocaba (UNISO), Sorocaba, SP Brazil; 9 São Paulo Fungal Group, São Paulo, Brazil

**Keywords:** Basidiomycetes, Brown-rot, Genome, Transcriptome, Proteome, CAZymes, Fenton reaction, Sugarcane by-products

## Abstract

**Background:**

Wood-decay basidiomycetes are effective for the degradation of highly lignified and recalcitrant plant substrates. The degradation of lignocellulosic materials by brown-rot strains is carried out by carbohydrate-active enzymes and non-enzymatic Fenton mechanism. Differences in the lignocellulose catabolism among closely related brown rots are not completely understood. Here, a multi-omics approach provided a global understanding of the strategies employed by *L. sulphureus* ATCC 52600 for lignocellulose degradation.

**Results:**

The genome of *Laetiporus sulphureus* ATCC 52600 was sequenced and phylogenomic analysis supported monophyletic clades for the Order Polyporales and classification of this species within the family Laetiporaceae. Additionally, the plasticity of its metabolism was revealed in growth analysis on mono- and disaccharides, and polysaccharides such as cellulose, hemicelluloses, and polygalacturonic acid. The response of this fungus to the presence of lignocellulosic substrates was analyzed by transcriptomics and proteomics and evidenced the occurrence of an integrated oxidative–hydrolytic metabolism. The transcriptomic profile in response to a short cultivation period on sugarcane bagasse revealed 125 upregulated transcripts, which included CAZymes (redox enzymes and hemicellulases) as well as non-CAZy redox enzymes and genes related to the synthesis of low-molecular-weight compounds. The exoproteome produced in response to extended cultivation time on Avicel, and steam-exploded sugarcane bagasse, sugarcane straw, and Eucalyptus revealed 112 proteins. Contrasting with the mainly oxidative profile observed in the transcriptome, the secretomes showed a diverse hydrolytic repertoire including constitutive cellulases and hemicellulases, in addition to 19 upregulated CAZymes. The secretome induced for 7 days on sugarcane bagasse, representative of the late response, was applied in the saccharification of hydrothermally pretreated grass (sugarcane straw) and softwood (pine) by supplementing a commercial cocktail.

**Conclusion:**

This study shows the singularity of *L. sulphureus* ATCC 52600 compared to other Polyporales brown rots, regarding the presence of cellobiohydrolase and peroxidase class II. The multi-omics analysis reinforces the oxidative–hydrolytic metabolism involved in lignocellulose deconstruction, providing insights into the overall mechanisms as well as specific proteins of each step.

**Supplementary Information:**

The online version contains supplementary material available at 10.1186/s13068-021-01945-7.

## Background

Wood-decay basidiomycetes are essential for the carbon cycle because of their highly specialized biomass degradation. Their metabolic systems include carbohydrate-active enzymes (CAZymes), but also non-CAZymes and other associated non-enzymatic compounds. This ability allows them to be potentially used for the production of value-added biocompounds derived from lignocellulosic biomass [1–3].

Traditionally, wood-decay basidiomycetes have been classified as brown-rot or white-rot based on the capacity to degrade plant cell wall components. Accordingly, brown rots degrade cellulose and hemicellulose while only modifying lignin. These two decay modes have been distinguished based on the reduction or absence of some enzymes, such as ligninolytic peroxidases (PODs) class II (manganese-, lignin- and versatile-peroxidases), as well as enzymes involved in cellulose degradation such as cellobiohydrolase (CBH), lytic polysaccharide monooxygenase (LPMO) and cellobiose dehydrogenase (CDH) [3, 4]. To compensate for the paucity of cellulolytic enzymes, some brown rots employ mechanisms for endoglucanase overproduction [5]. The lignocellulose degradation performed by brown-rot fungi involves chemical, biological and spatial relationships between fungal hyphae and the plant cell wall to perform a two-step mechanism: earlier lignocellulose oxidative (LOX) degradation mediated by Fenton reaction (H_2_O_2_ + Fe^2+^  → Fe^3+^  + OH**·**) followed by a late hydrolytic mechanism. Key requirements for Fenton systems include mechanisms for extracellular peroxide production and iron reduction, involving extracellular fungal enzymes and metabolites, to generate reactive oxygen species (ROS) [6–9].

Most brown-rot agaricomycetes belong to the order Polyporales Gäum. Within this order, most brown-rot species belong to the “Antrodia clade”, which includes the families *Dacryobolaceae* Jülich, *Fomitopsidaceae* Jülich, *Laetiporaceae* Jülich, and *Sparassidaceae* Herter, as well as a few unsolved groups [10]. *L. sulphureus* is considered a cosmopolitan species causing brown cubical heart rot in many deciduous and coniferous trees [11, 12]. It is known to produce metabolites with antioxidant and antimicrobial properties [13, 14] and natural dyes [15, 16] in addition to the potential for bioremediation of treated wood [17] and decolorization of textile effluents [11]. During wood decay, *L. sulphureus* causes higher polysaccharide weight loss than lignin loss [18]. This fungus has superior potential to produce cellulolytic and hemicellulolytic enzymes in comparison to other representative brown rots [19] and the enzymatic repertoire secreted in the presence of carboxymethyl-cellulose (CMC) was analyzed by mass spectrometry [20]. However, the potential for lignocellulose degradation has not been explored at multi-omics level.

Omics approaches allow a deep understanding of the biology of an organism, including its behavior during growth on complex plant biomass [21]. In this work, genome sequencing followed by transcriptomic and proteomic analysis provided a global understanding of the strategies employed by *L. sulphureus* ATCC 52600 in the degradation of lignocellulosic by-products derived from sugarcane and *Eucalyptus*. In addition, a commercial enzymatic cocktail supplemented with the *L. sulphureus* secretome was evaluated for saccharification of hydrothermally pretreated grass (sugarcane straw) and softwood (pine).

## Results

### Sequencing, annotation, and phylogenetic analysis of *L. sulphureus* ATCC 52600

The *L. sulphureus* ATCC 52600 genome sequence was assembled by a combination of paired-end (45,000,408 sequences) and mate-pair libraries (13,294,823 and 13,280,039 sequences), corresponding to 43.4 Mb (Table 1).

**Table 1:** Statistical information on the genome assembly of *L. sulphureus* ATCC 52600

*L. sulphureus* ATCC 52600	
Estimated coverage	125x
# contigs (> = 5000 pb)	428
# contigs (> = 10,000 pb)	375
# contigs (> = 25,000 pb)	275
# contigs (> = 50,000 pb)	213
# scaffolds	785
Total length (pb)	43,372,605
Largest contig (pb)	1,372,164
GC (%)	51.22
N50	211,056
N75	102,005
L50	53
L75	1129
Number of predicted genes	12,802

**Fig. 1:**
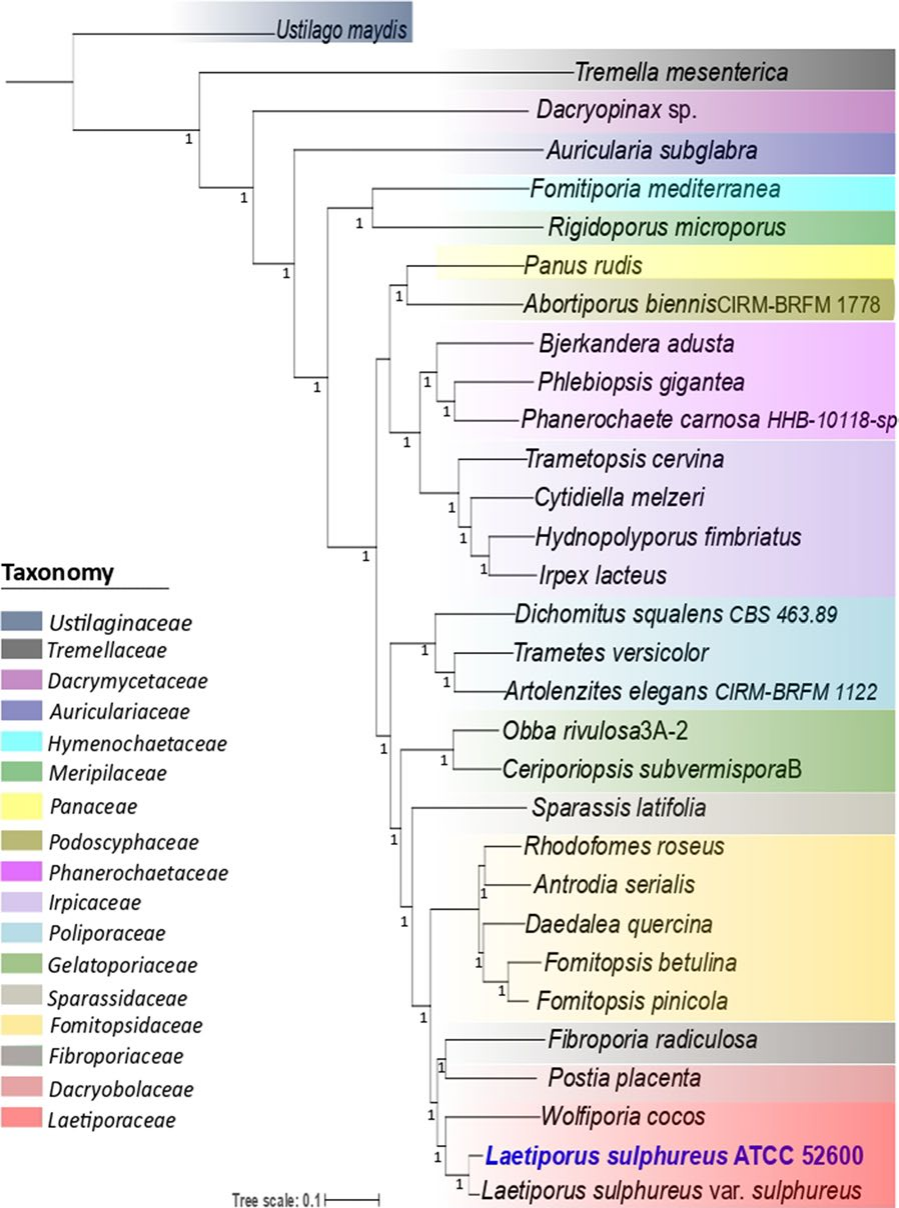
Phylogenomic analysis of *L. sulphureus* ATCC 52600 and related genera. The tree was built using the maximum likelihood (ML) method implemented in FastTree and WAG evolutionary models. A total of 601 single-copy ortholog genes from 31 genomes of basidiomycetes belonging to the order Polyporales were analyzed. Bootstrap values (1000 resamples) above 0.8

Genomic features were similar to *L. sulphureus* var. *sulphureus* v1.0 [22]. Comparative analysis showed the strains sharing 8419 clusters of orthologous genes, with 7724 single-copy genes, which accounted for 60% and 56% of all coding sequences for the strains ATCC 52600 and var. *sulphureus* v1.0, respectively. The phylogenomic analysis considering whole-genome information strongly supported monophyletic clades for all families within the order Polyporales (Fig. 1). The strain ATCC 52600 clustered with *L. sulphureus* var. *sulphureus* v1.0 and *Wolfiporia cocos* in the family *Laetiporaceae*, which, in turn, appears as a sister clade of *Fibroporiaceae* (*Fibroporia radiculosa*) and closely related to *Dacryobolaceae* (*Postia placenta*).

### *L. sulphureus* ATCC 52600 genome: non-enzymatic mechanism for biomass deconstruction and non-canonical brown-rot CAZymes

**Fig. 2:**
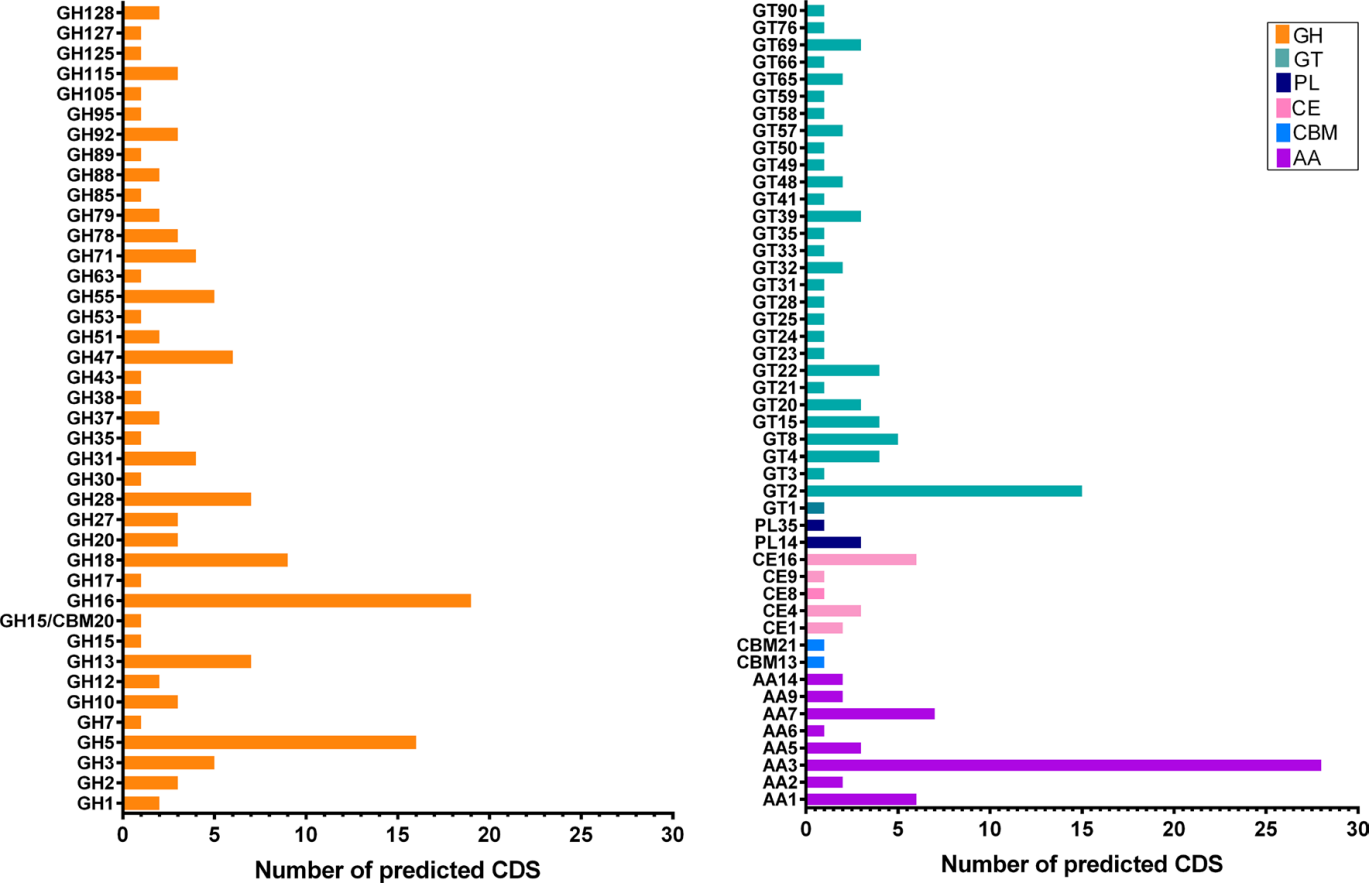
*L. sulphureus* ATCC 52600 CAZyme-coding genes. Genome profile representing the number of predicted genes encoding CAZymes. CAZy classes: GH: glycoside hydrolases, CBM: carbohydrate-binding module, CE: carbohydrate esterase, PL: polysaccharide lyase and AA: auxiliary activities

The CAZymes content was constituted of 271 modules, including 133 glycoside hydrolases (GH), 51 auxiliary activities (AA), 67 glycosyltransferases (GT), 13 carbohydrate esterases (CE), and 4 polysaccharide lyases (PL). Regarding carbohydrate-binding modules, CBM20 was found associated with GHs, in addition to the non-appended CBM13 and CBM21 (Additional file 1: Figure S1A). GHs comprised 49% of CAZymes, and the most abundant families were GH16 (19 members) and GH5 (16 members) (Additional file 1: Figure S1A). GHs repertoire for cellulose degradation also included endoglucanases (GH5), β-glucosidases (GH1, GH3), and one predicted cellobiohydrolase (GH7). A wide range of GHs associated with hemicellulose degradation such as xylan (GH10, GH43, GH115), glucans (GH16, GH55), and mannans (GH53) was identified, in addition to enzymes active on starch (GH13, GH15), pectin (GH28), chitin (GH18), and trehalose (GH37) (Fig. 2 and Additional file 2: Table S1).

The analysis showed 19% of the predicted CAZymes assigned to AAs (Additional file 1: Figure S1A). Among them, a large number of AA3 (28 members) are grouped into the subfamilies AA3_2 (25 aryl/glucose oxidases) and AA3_3 (3 alcohol oxidases). Also, 6 members of AA1 were identified and categorized into the subfamilies AA1_1 (3 laccases), AA1_2 (1 ferroxidase), and AA1_3 (2 laccase-like multicopper oxidases). Three AA5_1 glyoxal oxidases and one AA6 benzoquinone reductase were also identified. Moreover, two genes coding for AA2 PODs were predicted, as well as AA members acting on cellulose/hemicellulose, including seven AA7 glucooligosaccharide oxidases, two AA9 lytic polysaccharide monooxygenases (LPMO), and two AA14 LPMOs (Fig. 2 and Additional file 2: Table S1).

The most prevalent CE family was CE16 (6 acetyl esterases), in addition to CE4 (2 chitin deacetylase and 1 acetyl xylan esterase), CE1 (1 acetyl xylan esterase and 1 carboxylesterase), CE8 (1 pectin methylesterase), and CE9 (N-acetylglucosamine deacetylase). Three PL14 and one PL35 were also identified. The most abundant GT family was GT2 encompassing 15 members, followed by GT8 (5 members), GT4 (4 members), and GT15, GT20, GT21, GT39, and GT69 (3 members each) (Fig. 2, Additional file 1: Figure S1A and Additional file 2: Table S1). Additionally, a wide diversity of genes involved in Fenton reaction and oxidative mechanisms was identified in the *L. sulphureus* ATCC 52600 genome, including alcohol dehydrogenases, aldo–keto reductases, catalases, ferroxidase, cytochrome P450, peroxidases not assigned to CAZy domains, oxidoreductases, oxalate decarboxylase, and hydroquinone dehalogenases, the last two involved in the production of low-molecular-weight compounds (LMW) (Additional file 1: Figure S1A and Additional file 2: Table S1).

### *L. sulphureus* ATCC 52600 displays a broad-range carbohydrate metabolism and slow glucose consumption

The presence of transporters for different carbohydrates such as glucose, mannose, and trehalose in the genome motivated further analysis of the *L. sulphureus* ATCC 52600 primary metabolism (Additional file 2: Table S1). The strain was able to grow on mono-, di- and polysaccharides (Additional file 1: Figure S2A), and faster colony growth was verified on pectin, followed by galacturonic acid, xylan, arabinose, and galactomannan (Additional file 1: Figure S2B). The growth analysis in liquid medium with glucose showed an extended lag period, with glucose consumption starting after 48 h of cultivation, which then decreased at a slow rate to around 40% at 168 h of cultivation (Additional file 1: Figure S2C). These data raised questions about the biological behavior of this basidiomycete growing on complex carbon sources in terms of protein expression and secretion, especially because of the presence of some non-canonical brown-rot CAZymes such as CBH and AA2 peroxidase in the genome.

### Transcriptomic analysis reveals the early response of *L. sulphureus* ATCC 52600 for the deconstruction of sugarcane bagasse

A total of 10,015 transcripts were identified with at least one transcript per million (TPM), with 6920 sequences presenting statistical significance. Differential expression analysis revealed 1120 up- and 1455 downregulated genes, which included 96 CAZy transcripts and a set of 159 genes involved in redox metabolism (non-CAZy) (Additional file 1: Figure S1B and Additional file 2: Table S2). Among the CAZymes, 63 and 33 genes were up- and downregulated, respectively, with GHs comprising 60% of the upregulated genes. Five and 17 genes with predicted cellulolytic and hemicellulolytic functions, respectively, were identified among the upregulated CAZy transcripts, including glucanases (GH5, GH16, GH55, and GH71), β-glucosidases (GH1 and GH3), α-xylosidase (GH31), α/β-mannosidases (GH2 and GH47), and α-/β-galactosidases (GH27, GH35, and GH71). In addition, several genes with predicted activity on starch (GH13 and GH31), chitin (GH18 and CE4), and pectin (GH28, GH78, and GH105) were upregulated, whereas transcripts predicted for xylan-active xylanase (GH30) and β-xylosidase (GH43) were downregulated.

**Fig. 3:**
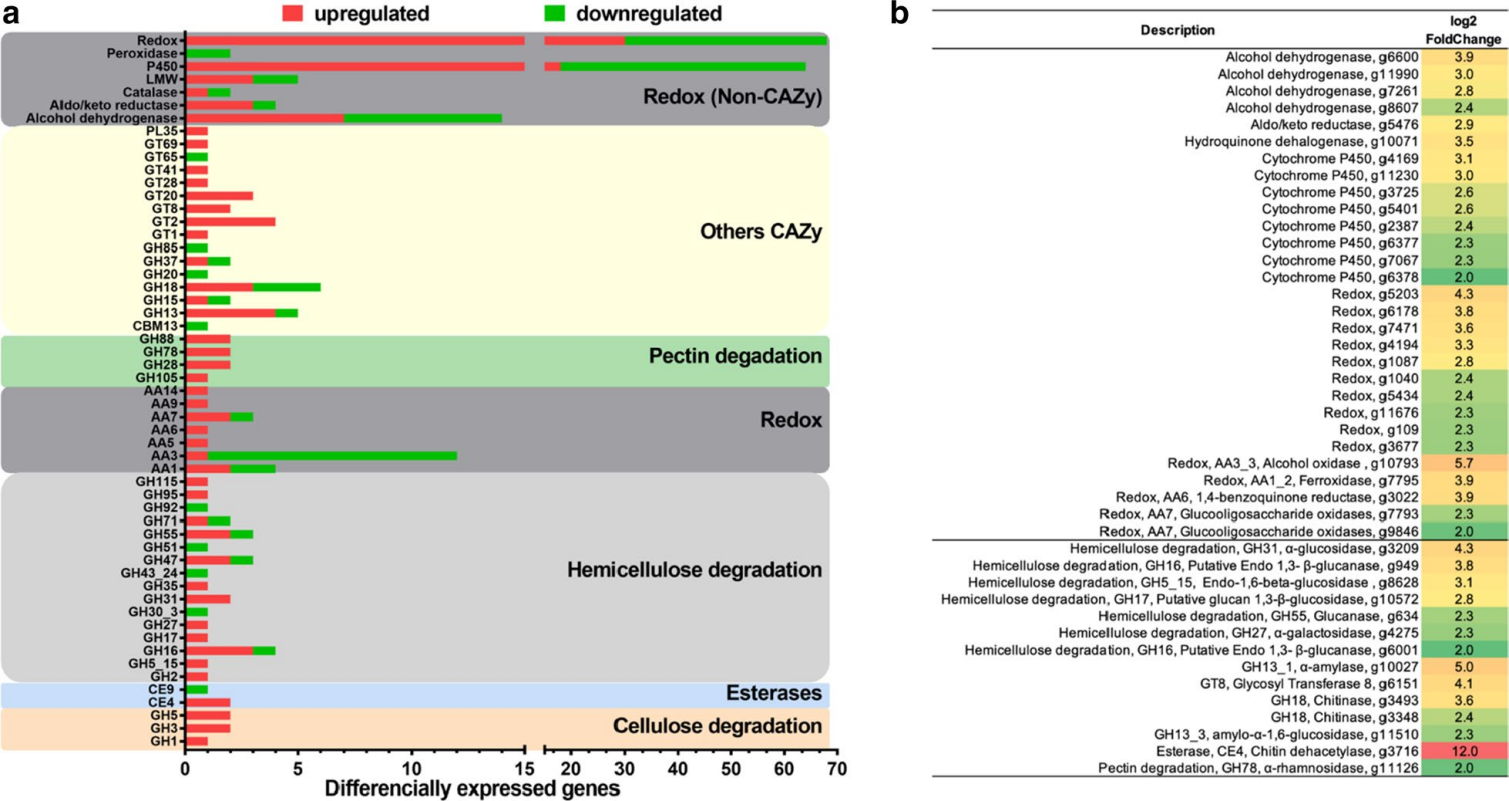
Differentially expressed genes of *L. sulphureus* ATCC 52600 cultivated on sugarcane bagasse. **a** Up- and downregulated CAZyme genes and selected redox genes (non-CAZy) grouped according to their predicted function. **b** Highly expressed transcripts related to the *L. sulphureus* CAZy arsenal and oxidative mechanism (log_2_-fold change ≥ 2). CAZy classes: GH: glycoside hydrolases, CBM: carbohydrate-binding module, CE: carbohydrate esterase, PL: polysaccharide lyase, and AA: auxiliary activities

Among the AAs, two AA1_1 laccases and one AA1_2 ferroxidase were upregulated; AA3 members such as the 11 members of the subfamily AA3_2 were downregulated, while one AA3_3 alcohol oxidase was upregulated. In addition, two AA7 glucooligosaccharide oxidases, one AA6 benzoquinone reductase, one AA9 LPMO, and one AA14 LPMO were upregulated (Fig. 3a and Additional file 2: Table S2).

A set of genes encoding non-CAZy enzymes and proteins with a predicted function in the oxidative mechanism and Fenton reaction were also regulated, corresponding to 159 transcripts, out of which 62 were up and 97 downregulated (Fig. 3a and Additional file 2: Table S2). The importance of this mechanism became clearer when the regulation of individual genes was analyzed, i.e., among the high upregulated transcripts (log2-fold change ≥ 2, *n* = 43), 67% were associated with oxidative mechanisms, including both AAs and non-CAZymes. The remaining CAZymes (33%) were mostly hydrolases including miscellaneous hemicellulases (acting on glucan, mannan, galactan), amylases, pectinase, and chitinases. Remarkably, the top upregulated transcripts included a series of AA oxidoreductases belonging to the families AA3_3, AA1_2, AA6, and AA7, as well as non-CAZy oxidoreductases, dehydrogenases, cytochrome, and enzymes involved in LMW metabolism. In turn, transcripts of predicted cellulose- or xylan-active enzymes were absent (Fig. 3b).

### Proteins secreted by *L. sulphureus* ATCC 52600 during cultivation on pretreated plant biomass

The exoproteomes of *L. sulphureus* ATCC 52600 cultivated on lignocellulosic biomass were analyzed by tandem mass spectrometry. A total of 3328 spectra were identified, accounting for 112 proteins. This set of proteins was composed of 42 CAZymes, 8 peptidases/proteases, 8 non-CAZy oxidoreductases, 7 esterases, 5 dehydrogenases, 32 miscellaneous proteins/domains (denominated “others”), and 10 hypothetical proteins of unknown function (Additional file 1: Figure S1C and Additional file 2: Table S3). Of note, most of the proteins identified were predicted with SP.

**Fig. 4:**
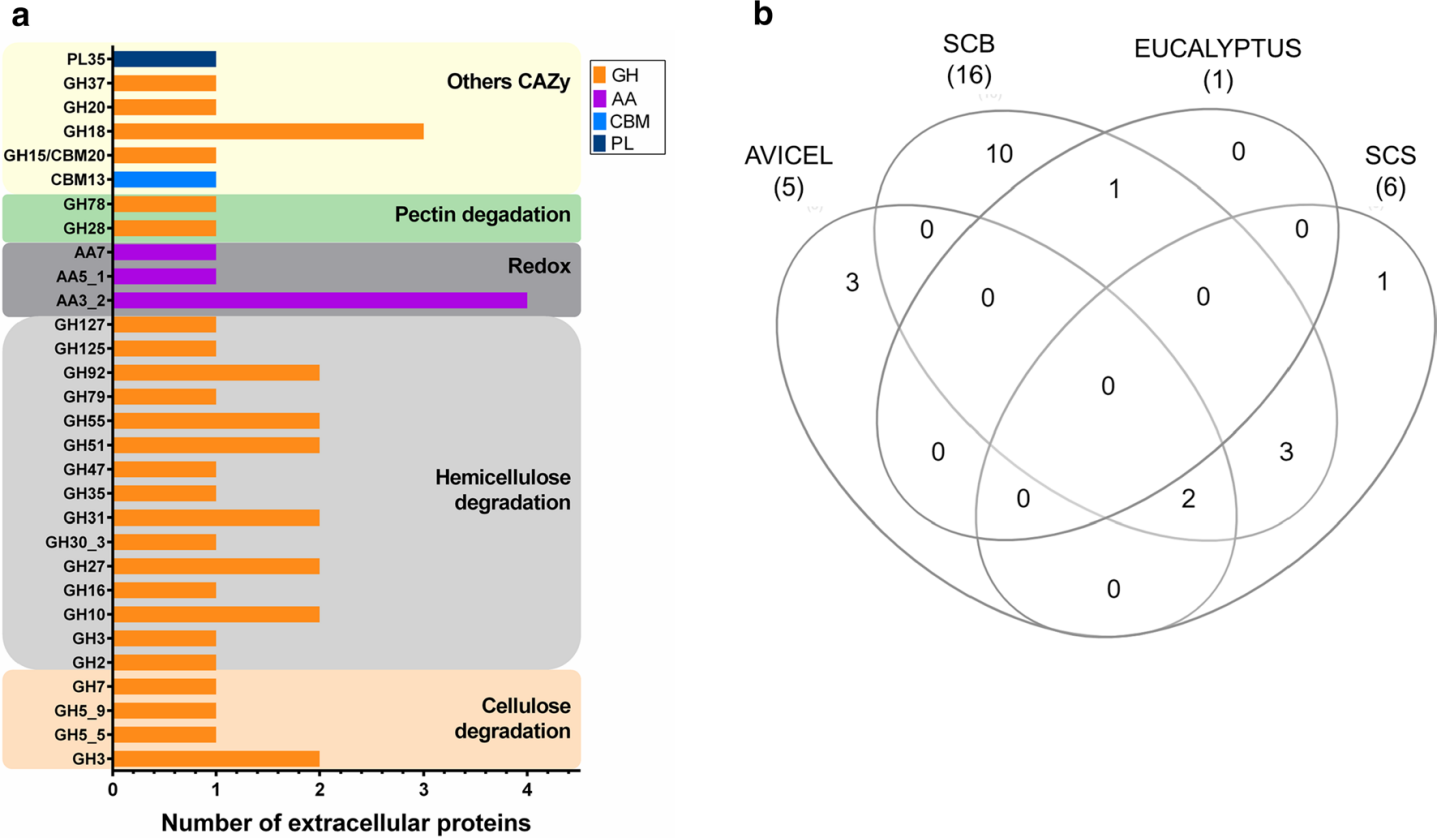
Overview of CAZymes identified in the *L. sulphureus* ATCC 52600 secretomes. **a** CAZy classes: GH: glycoside hydrolases, CBM: carbohydrate-binding module, CE: carbohydrate esterase, PL: polysaccharide lyase, and AA: auxiliary activities. **b** Venn diagrams grouping upregulated CAZymes relative to glucose. *SCB* sugarcane bagasse, *Eucalyptus:* *Eucalyptus grandis* residue, *SCS* sugarcane straw

GHs were predominant among the secreted CAZymes, accounting for 80% (34 members), followed by 6 AA members (Additional file 1: Figure S1C and Additional file 2: Table S3). Most of the GHs (62%) corresponded to enzymes with predicted activity on hemicellulose, while 5 were cellulose-active GHs. In addition, GHs with predicted activity on pectin, starch, chitin, and trehalose, as well as one PL35 alginate lyase were also secreted (Additional file 2: Table S3). Overrepresented GH families corresponded to GH18 with predicted chitinolytic activity (g5150, g834, and g10854), and GH3 represented by β-glucosidases (g2032 and g11777) and β-xylosidase (g7390). The secreted AAs array consisted of AA3 aryl alcohol oxidases (g5677, g5675, g5206, and g10342), AA5 glyoxal oxidase (g4370), and AA7 glucooligosaccharide oxidase (g9758) (Fig. 4a).

The highest number of CAZymes was identified in the secretome produced on SCB (40 proteins), followed by 33, 30, 27, and 16 proteins identified on *Eucalyptus*, Avicel, SCS, and glucose, respectively. Differences in the CAZyme arsenal produced by *L. sulphureus* ATCC 52600 for lignocellulose degradation became evident when comparing the secretomes (Fig. 4b and Additional file 2: Table S3). A total of 16 CAZymes were secreted in all conditions, indicating constitutive secretion. The secretome produced on SCB showed the highest number of upregulated CAZymes, comprising 16 hits, out of which 10 were exclusively found in this condition. These hits corresponded to exo-type enzymes mainly related to hemicellulose degradation such as GH3 β-xylosidase (g7390), GH35 β-galactosidase (g11423), and GH47 α-mannosidase (g10983), along with GH3 β-glucosidase (g11777) and AA7 oxidoreductase (g9758). Moreover, the secretomes produced on SCB and SCS showed common upregulation of GH3 (g2032), AA3_2 (g5675), and GH20 (g8819). Interestingly, some enzymes with basal constitutive secretion in the secretome produced on glucose such as GH7 CBH (g8442), GH92 α-1,2-mannosidase (g9634), and GH18 chitinase (g834) were upregulated on Avicel. Additionally, one xylanase (g4476) was upregulated on all polymeric substrates, and one AA3_2 aryl alcohol oxidase (g10342) was exclusively upregulated on SCS. Despite the high number of proteins secreted on *Eucalyptus*, only one β-L-arabinofuranosidase (g6508) was upregulated in this condition as well as on SCB. Another remarkable characteristic was the absence of AAs in the secretome produced on Avicel. Finally, some proteins among the 17 non-CAZy classified as “others” were upregulated at least in one condition, mostly on SCB. Prominent among them were galactose oxidase (g7548) and another oxidoreductase (g9473), both showing upregulation on Avicel, SCB, and *Eucalyptus* (Additional file 2: Table S3).

### Performance of the *L. sulphureus* ATCC 52600 secretome on biomass conversion

Enzymatic activity profiles were evaluated on the *L. sulphureus* ATCC 52600 secretomes and most of the identified activities were found at higher levels on SCB. Activities were detected on arabinoxylan, β-glucan, starch, and xylan from beechwood (Additional file 1: Figure S3), corroborating the enzymes identified in the secretomes (Additional file 2: Table S3). The secretome produced on SCB was then applied for supplementing commercial enzymatic cocktails in the saccharification of pretreated lignocellulosic biomass such as sugarcane straw (grass) and pine (softwood).

**Fig. 5:**
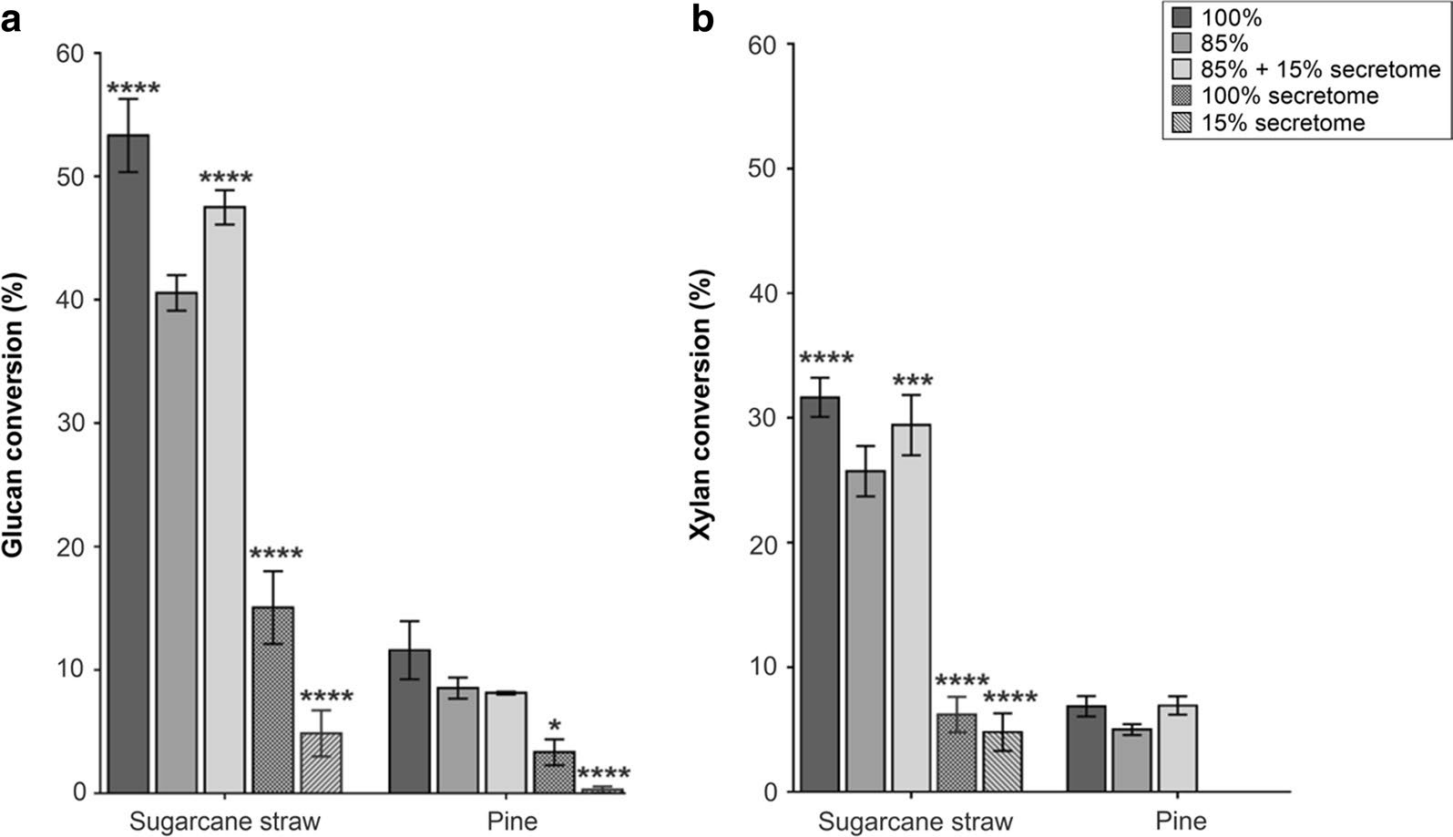
Saccharification of hydrothermally pretreated sugarcane straw and pine. **a** Glucan and **b** xylan enzymatic conversion. The reaction was performed with a mixture of Celluclast®: glucosidase from *Aspergillus niger* (5:1 w/w) supplemented with *L. sulphureus* ATCC 52600 secretome produced on SCB. Total protein load corresponds to 15 FPU/g of substrate (100%). Reactions containing 2% (w/v) solids load in 50 mM sodium acetate pH 5.0 were incubated for 48 h at 50 °C. Data correspond to mean values and standard deviations of four triplicates. Significance was analyzed using two-way ANOVA with Tukey’s test relative to the reference reaction "85% Celluclast" (95% confidence interval) and is indicated as follows: *p < 0.05, **p < 0.01, ***p < 0.001

Glucan conversion obtained with sugarcane straw ranged from 40 to 55% by using the commercial cocktail at 85% or 100% enzyme load, respectively. Replacing 15% of the commercial cocktail with the secretome produced on SCB increased the glucan conversion by 7% (Fig. 5a). In turn, no differences were observed in the xylan conversion, which was around 30% (Fig. 5b). Saccharification of pine lignocellulose was noticeably less efficient for both glucan (12%) and xylan (around 8%) conversion, and unresponsive to the enzymatic supplementation with the *L. sulphureus* secretome.

## Discussion

### *L. sulphureu*s ATCC 52600 genome does not resemble typical brown-rot fungi

Genomic sequencing of filamentous fungi followed by transcriptomic and proteomic approaches has been widely employed to understand the strategies of microorganisms to degrade plant biomass [23–29]. Overall, the *L. sulphureus* ATCC 52600 genome revealed only subtle differences compared to the previously sequenced *L. sulphureus* var. *sulphureus* v1.0 [22], indicating that the strains might have undergone some changes in their ecological niches to shape their genomes to the environmental conditions. Our phylogenetic analysis, providing high resolution on the evolutionary history of organisms by considering whole-genome information [29], complements the previous phylogeny of the order Polyporales [10]. The phylogenetic tree (Fig. 1) strongly supports monophyletic clades for the families within the order Polyporales. *L. sulphureus* ATCC 52600 clusters with *L. sulphureus* var. *sulphureus* v1.0 and *W. cocos* giving further support to the existence of the family *Laetiporaceae* Jülich, as previously proposed [10] and currently present in Mycoguide, but retrieved as an invalid name in MycoBank and Index Fungorum.

The genomic CAZyme content in both *L. sulphureus* strains and the closely related brown-rot Polyporales shows a typical number of GHs, CEs, PLs, and GTs compared to *W. cocos*, *P. placenta* and *F. radiculosa*, whereas a lower number of CAZymes, particularly GHs, were observed in comparison with *Fomitopsis pinicola* (Fomitopsidaceae). In turn, *L. sulphureus* ATCC 52600 shows a higher AA content than in the other genomes [9]. Additionally, the genome presents several similarities with other brown-rot genomes associated with evolutionary reductions and losses in key enzymes involved in biomass breakdown, especially cellulases and lignin-modifying enzymes [3]. Accordingly, it presents a reduced number of genes coding CAZymes from the families GH1, GH3, GH5, GH7, GH10, AA9, and CE1 along with the absence of GH6, GH11, AA3_1, CBM1, and CE15 (Additional file 2: Table S1).

Considering these reductions or absences, other enzymes may also be necessary to achieve an effective breakdown of cellulose and hemicellulose, such as the AA9 and AA14 LPMOs. AA9s perform oxidative cleavage on cellulose and other glucans with great importance in lignocellulose degradation [30], presenting an average number of 3 genes in Polyporales genomes [3, 26]. The recently established family AA14 also groups LPMOs that are widespread in fungi. Within the order Polyporales, there are 4.5 and 2.5 AA14 coding genes on average in white and brown-rot genomes, respectively [31]. This reduction pattern can also be observed for other gene reductions associated with the brown-rot lifestyle evolution. One characterized AA14 member from the white-rot *Pycnoporus coccineus* presents oxidative activity on xylans of xylan-coated cellulose fibers [31], and shares 48.8% identity with the *L. sulphureus* ATCC 52600 AA14 LPMO (Additional file 1: Figure S4).

Regarding the enzymes involved in the oxidative mechanism, AA3_1 CDHs are absent in *L. sulphureus*, as verified in *P. placenta*, *W. cocos,* and *F. pinicola* [32]. In turn, a large number of genes coding for AA3_2 (aryl alcohol oxidase and glucose 1-oxidase) and AA3_3 (alcohol oxidase) was identified, and the products H_2_O_2_ (reduction of oxygen by oxidases) and hydroquinones (reduction of quinones) can support other enzymes that are important for lignocellulose deconstruction [33]. Similarly, AA5_1 glyoxal oxidases and AA6 benzoquinone reductase, which are also involved in Fenton reagents generation [34–36], were identified (Additional file 2: Table S1). Notably, the absence of CDH may also suggest the presence of other redox partners for the AA9 and AA14 LPMOs, such as AA3_2 flavoenzymes [37] and GMC oxidoreductases, among others [38], or the peroxide production might be driving LPMOs reaction [39].

The *L. sulphureus* genome also revealed some distinctions in the lignocellulolytic repertoire. For example, the well-known lack of cellulases in brown-rot fungi is generally attributed to a reduced number of GH6/GH7 CBHs [32, 40, 41], which are absent in brown-rot Polyporales [42]*.* Our sequencing, however, identified one putative GH7 CBH (g8442) in the *L. sulphureus* ATCC 52600 genome, in accordance with a GH7 CBH previously identified in the secretome of *L. sulphureus* growing on CMC [20]. Sequence analysis shows these enzymes sharing more than 90% identity, and the phylogeny using predicted and characterized fungal CBHs reveals 65% similarity with other fungal CBHs (Additional file 1: Figure S5). Additionally, analysis of 42 fungal genomes indicates that brown rots generally have a reduced number of GH45, in a 3:1 ratio in comparison with white rots [9]. Our initial search parameters identified one putative GH45 (g10751), coinciding with a GH45 (ID 174,393) previously identified in the *L*. *sulphureus* secretome [20]. These sequences share 92.5% identity; having an expansin domain predicted by InterPro v.78.1 [43], despite the previous classification as GH45 class C [20]. Expansins are closely related to GH45 endoglucanases and have been widely found in brown-rot strains [9], playing an important function in reducing biomass recalcitrance, consequently increasing the deconstruction of lignocellulose in synergism with cellulases [44].

Lignin degradation and the importance of different lignin-active enzymes in brown rots is a matter of debate, but it is widely recognized that brown rots present a reduced number of laccases and absence of PODs class II in comparison with white-rot strains [8, 45, 46]. *L. sulphureus* ATCC 52600 has AA1_1 and AA1_3 laccases, similar to *F. pinicola*, *P. placenta,* and *W. cocos* [47]*.* Additionally, 13 predicted PODs were identified in the *L. sulphureus* ATCC 52600 genome, and two of them with predicted AA2 domain. InterPro annotation classified one of them as an intracellular POD class I, while the other (g11846) was classified as a fungal ligninase/POD class II with a predicted SP. BLAST search retrieved 87% and 66% identity with PODs class II from *L. sulphureus* var. *sulphureus* v1.0 and *W. cocos* MD-104SS10 v1.0, respectively. POD class II has been reported as a single copy in *P. placenta*, *W. cocos* and *F. pinicola* genomes [32], and the *P. placenta* peroxidase (Ppl44056) was classified as a basal peroxidase, not closely related to LiP and MnP [48]. Laccases in Polyporales are multigenic [49] and have been characterized as functional enzymes in *P. placenta* and *F. pinicola* [50–52]*,* playing a role in wood decay performed by *P. placenta* [50]. Significant lignolysis has been observed in *Gloeophyllum trabeum* (Gloeophyllales) and *P. placenta* without considering the involvement of PODs class II [6, 53]. Nevertheless, the biological importance or the precise role of these PODs II found specifically in *L. sulphureus* and other closely related brown rots are uncertain since these enzymes have not been characterized to date.

### Insights into the *L. sulphureus* ATCC 52600 biomass deconstruction mechanism

Several omics studies analyzing brown-rot fungi with significant taxonomic and niche distances such as *W. cocos, F. radiculosa, P. placenta, G. trabeum,* and *Serpula lacrymans* (Boletales), cultivated in different conditions show the common presence of a two-step mechanism involved in biomass deconstruction [8, 25, 48, 54–60]. The initial oxidoreductive step is estimated to persist for 48 h [8], which can be correlated with both the observed slow growth of *P. placenta* in cellulose and spruce [56] and the *L. sulphureus* growth and glucose consumption in liquid medium (Additional file 1: Figure S2C).

The transcriptome data of a short cultivation period reveal a series of upregulated genes related to the oxidative mechanism, probably induced by the recalcitrance of the non-pretreated sugarcane bagasse (Fig. 3 and Additional file 2: Table S2). The most upregulated transcripts include alcohol dehydrogenase, cytochrome P450, aldo/keto reductases, and redox genes involved in the generation of hydrogen peroxide, while hydroquinone dehalogenase is involved in hydroquinone production that initiates Fenton reaction by carrying Fe^3+^ [61]. Moreover, the presence of AA6 quinone reductases suggests that this enzyme takes part in the quinone redox cycle supporting Fenton chemistry, as previously observed in *P. placenta* [48], while also playing a role in the detoxification process [5]. Such observations are consistent with a biodegradative role of Fenton chemistry occurring during early cultivation of *L. sulphureus* on SCB, as verified in other brown-rot transcriptomes [9, 62, 63].

Regarding CAZymes (Fig. 3a and Additional file 2: Table S2), previous brown-rot transcriptomic studies similarly revealed a small set of cellulases and hemicellulases with predicted activity on glucans and mannans [8, 48]. The upregulation of some cellulases and hemicellulases supports the existence of inducing mechanisms, which may depend on substrate exposure and availability but operating differently than reported for white-rot basidiomycetes or ascomycetes [56]. Additionally, the upregulation of two AA1 laccases indicates an ability to partially oxidize lignin. On the other hand, two other AA1 laccases, as well as two non-CAZy peroxidases were downregulated, so the importance of ligninases for this fungus remains unclear (Fig. 3a and Additional file 2: Table S2). Transcripts of AA9 and AA14 LPMOs were upregulated, but not secreted, corroborating the concept of LPMOs being produced by fungi during early biomass degradation [62, 64, 65]. Our data indicate that *L. sulphureus* adopts mechanisms to integrate enzymatic and non-enzymatic systems at initial stages of brown-rot decay, as previously reported in *G. trabeum* [65]. Of note, the biological importance of LPMOs for brown-rot fungi remains unclear since their secretion has only been identified in *G. trabeum* growing on lignocellulose [48].

Furthermore, the growing of *L. sulphureus* on pectin and the upregulation of pectinases transcripts (Additional file 1: Figure S2A and B and Additional file 2: Table S2) support the mechanism in which pectin degradation also occurs in the early stage of degradation as previously observed in *P. placenta* and *G. trabeum*. Pectin removal by pectinases may facilitate the access of other enzymes to the plant cell wall components [8, 56, 57].

In contrast to the transcriptome, our secretome data (7-day cultivation) represents a late hydrolytic decay profile [56], which is supported by the absence of AAs in the secretome produced on Avicel. A core set of constitutive CAZymes was identified, comprising some GHs with predicted activity on cellulose and a wide diversity of GHs acting on glucans, xylan, mannans, trehalose, starch, and chitin (Additional file 2: Table S3). Apart from the xylan-active enzymes, the hemicellulase set is very similar to the profile observed in the transcriptome. This complete set of hemicellulases found in brown rots [56] allows the fungus to obtain energy sources from diverse substrates, providing an increase in survival capability under different environmental conditions.

Additionally, regulatory mechanisms may take place after the sensing and transport of inducers, resulting in the secretion of a repertoire of CAZymes targeted to substrate degradation. In that sense, differences in the enzymatic arsenal can be observed in the secretomes of *L. sulphureus* produced on grass and wood-derived substrates, which typically present different compositions [66, 67]. The secretome produced on SCB showed the highest diversity of upregulated proteins probably due to substrate recalcitrance and pretreatment characteristics [68, 69].

Endoglucanases are poorly secreted by *L. sulphureus,* apparently playing a minor role in cellulose degradation, despite the importance of processive endoglucanases in brown rots [70]. Two GH3 β-glucosidases are upregulated on SCB and notably the GH7 CBH undergoes upregulation exclusively on Avicel. These data, in addition to the basal secretion of some CAZymes commonly found in all substrates, show that CBH is inducible and it is not under carbon catabolite repression, as verified for the endoglucanase from *G. trabeum* [71] or cellulases from *P. placenta* [56]*.* However, the gene encoding GH7 CBH is not differentially expressed (transcriptome—early stage), and the secretion of endoglucanases and β-glucosidases as well as oxidative agents, may compensate for that low expression in early stages [4, 6].

In addition to the constitutive hemicellulases, a diversity of enzymes acting on glucan and mannan (mannosidases and α-galactosidases) were upregulated at both early and late response to biomass degradation (Additional file 2: Tables S2 and S3) corroborating a natural preference of brown rots for softwoods [1, 3, 7, 59]. There is evidence that hemicellulose loss progresses faster than cellulose loss in coniferous wood decay performed by *G. trabeum*, *F. pinicola, W. cocos* and *L. sulphureus* [72, 73]. Additionally, our secretome data show that *L. sulphureus* targets hemicellulose as part of the hydrolytic late response. Several enzymes active on xylan, the main hemicellulose in grasses [74], were secreted by *L. sulphureus*, i.e., one GH10 xylanase is upregulated on Avicel and SCB, while the production of another GH10 xylanase is constitutive. Also, one β-xylosidase is upregulated on SCB, while two α-L-arabinofuranosidases are widely secreted on the polymeric substrates (Additional file 2: Table S3). Moreover, transcripts of other arabinoxylan-degrading enzymes do not show early upregulation; rather, one GH30 xylanase and one GH51 arabinofuranosidase are downregulated (Additional file 2: Table S2). This result shows that *L. sulphureus* can adapt the metabolism to the degradation of a grass substrate, despite other brown rots from the Antrodia clade have been reported to be inefficient in the degradation of corn stalk [75].

Despite the ability of *L. sulphureus* to grow on xylan and galactomannan (Additional file 1: Figure S2A and B), in addition to the presence of some cellulases and several hemicellulases in the late-response secretome (Additional file 2: Table S3), biomass conversion was low, especially for softwood (Fig. 5). The lack of mannanases in the commercial cocktail [76] and the SCB secretome may be a possible explanation for the ineffective degradation of the pine biomass. Indeed, brown-rot fungi are known to grow and modify pine and other softwoods [73, 77, 78]; however, softwood is among the most recalcitrant lignocellulosic substrates for enzymatic processes, requiring severe pretreatment conditions as well as higher enzyme doses than hardwood or grass substrates [79]. Lignin content, larger amounts of extractive components, and smaller pore size are additional characteristics that may be further contributing to the poor degradation of this lignocellulose [80].

**Fig. 6:**
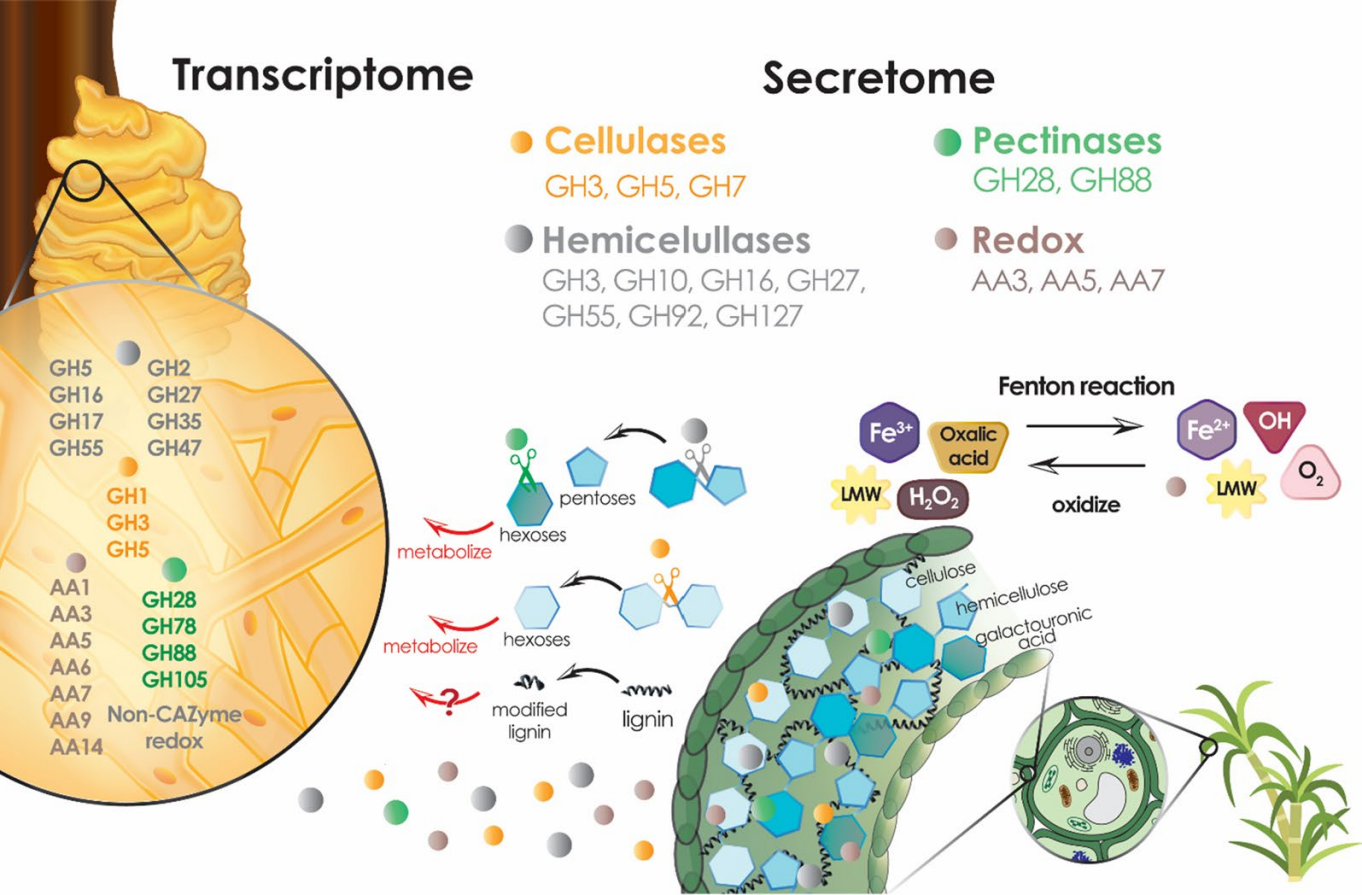
Biomass degradation mechanism from *L. sulphureus* ATCC 52600. Multi-omics analysis showing the range of CAZymes induced in response to sugarcane lignocellulose. The scheme represents the main CAZymes found in the transcriptome (in the basidium stem) and secretome analysis responsible for lignocellulose deconstruction and lignin modification by the oxidative mechanism, involving CAZymes, low-molecular-weight (LMW) compounds, and Fenton reaction. In parallel, monomers released from holocellulose are metabolized, unlike lignin, which remains partially degraded

In Fig. 6**,** an overview of the *L. sulphureus* strategies for biomass deconstruction is proposed based on our multi-omics data. Our results are consistent with a temporal two-step oxidative–hydrolytic mechanism for the degradation of lignocellulose, while also demonstrating that this fungus does not resemble typical brown-rot fungi in many aspects, thus contributing to the weak dichotomy between white- and brown-rot strains, as previously proposed [26]. Additional data applying biological approaches such as gene deletion and analysis of wood decay, as well as biochemical characterization of the enzymes would contribute to further address this question.

## Conclusions

Genome sequencing and analysis of expression and secretion patterns contributed to elucidate the mechanism involved in lignocellulose degradation by *L. sulphureus* ATCC 52600. In many aspects, this brown-rot fungus presents similarities with other model brown rots, while not resembling typical brown rots, especially due to the notable presence of cellobiohydrolase and POD class II. The transcriptomic analysis using highly recalcitrant biomass at a short cultivation period demonstrated the presence of early oxidative response, as well as other hallmarks of an early response such as the upregulation of pectinases and oxidative enzymes, including LPMOs. The late response was evaluated by proteomic analysis of secretomes produced on cellulose, and lignocellulose from grass (sugarcane bagasse and straw), and hardwood (*Eucalyptus*). Overall, the secretome profiles showed a common set of CAZymes in different conditions, with only subtle differences in the secretion of specific enzymes. Some cellulases displayed constitutive secretion while a more complex regulatory mechanism may be occurring for enzymes acting on xylan degradation. Another remarkable characteristic is the absence of AAs in the degradation of crystalline cellulose, but not in the degradation of lignocellulosic substrates.

## Methods

### Strain maintenance

*L. sulphureus* ATCC 52600 was purchased from Fundação André Tosello (CCT 4694). The strain was routinely maintained on solid media composed of 20 g/L malt extract and 2 g/L yeast extract and incubated for 7–10 days at 30 °C.

### DNA extraction and sequencing

DNA extraction from mycelia was performed using phenol–chloroform, followed by RNAse treatment. High-quality DNA was obtained using the DNeasy Kit (Qiagen). Three Illumina libraries were constructed, a paired-end library with a 300-bp insert and two mate-pair libraries with 5–7 and 8–11 kb, according to the manufacturer's instructions. The libraries were sequenced on an Illumina HiSEq 2500 platform.

### Genome assembly and annotation

Paired-end and mate-pair reads (2 × 100pb) were filtered by quality and presence of adaptors using Trimmomatic v0.32 [81] and NextClip [82] default parameters, respectively. The genome was de novo assembled using Velvet v.1.2.10 [83] with kmer = 55. The resulting assembly was scaffolded by SSPACE v3.0 [84] and the mate-pair reads, and further refined by Pilon version 1.16 [85]. The completeness of the genome was assessed using Benchmarking Universal Single-Copy Orthologs (Busco) [86] and the prediction was performed using BRAKER1 [87], which applies GeneMart-ET and AUGUSTUS along with RNA-seq alignments for gene prediction. Predicted protein sequences were functionally annotated by searching for homologous sequences in the SwissProt [88], EggNOG [89], and Pfam [90] databases. Signal peptides (SP), transmembrane regions, and ribosomal genes were predicted using SignalP v.4.0 [91], TMHMM [92], and ITSx using fungal models, respectively. Carbohydrate transporters were identified and classified according to the PFAM 00,083.21 [29] and enzymes associated with LMW metabolism were classified using Gene Ontology (http://geneontology.org). Comprehensive analysis of CAZymes was carried out using HMM-based dbCAN v.8 [93], HMMER (E-Value < 1e-15, coverage > 0.35), DIAMOND (E-Value < 1e-102), and Hotpep (Frequency > 2.6, Hits > 6).

### Phylogenetic analysis

The phylogenomic relationship of *L. sulphureus* ATCC 52600 and its closest described relatives of the family *Laetiporaceae* was determined based on orthologs single-copy genes using FastOrtho tool (https://github.com/olsonanl/FastOrtho). The protein sequences of each 601 single-copy orthologous genes present in 31 basidiomycete genomes closely related to the family *Laetiporaceae* and *L. sulphureus* ATCC 52600 were aligned by Mafft v.7.299 [94] and the resulting individual alignments were concatenated to create a supermatrix using FASconCAT-G v.1.02 [95]. Evolutionary distance was inferred using maximum likelihood with RAxML v.8.2.0 [96], implementing PROTGAMMAWAG model and performing 1000 bootstrap replicates to evaluate the reliability of the reconstructed phylogenetic tree.

### Cultivation conditions for transcriptome analysis

Pre-inoculum, consisting of 15 discs (8 mm diameter) of *L. sulphureus* ATCC 52600 pre-cultivated on agar plates, was inoculated into 100 mL of liquid medium and incubated under 180 rpm for 7 days at 30 °C. Mycelia were then filtered and washed with water and transferred to liquid medium containing 1.0 g of in natura sugarcane bagasse and 100 mL of medium pH 7.0 composed of 6 g/L (NH_4_)_2_SO_4_, 1 g/L KH_2_PO_4_, 1 g/L KCl, and 1 g/L MgSO_4_. Cultivation was performed under 180 rpm for 24 h at 30 °C. Mycelia and substrate mixtures were collected by filtration, washed with sterile water, manually dried in filter paper, and stored at -80 °C before RNA extraction. Mycelium from the pre-inoculum was used as a standard before induction (T_0_).

### RNA extraction and sequencing

The mycelium was ground with liquid nitrogen and total RNA extraction was performed with mirVana™ Total Isolation Kit (Thermo Fisher), according to the manufacturer's instructions. The resulting solution was treated with DNAse (DNA-Free RNA Kit, Zymo Research) and purified with RNeasy Kit (Qiagen), and quality was verified using RNAnano Bioanalyzer 2100 chip (Agilent). cDNA libraries were prepared according to the manufacturer's instructions and sequenced on the Illumina HiSEq 2500 platform.

### Bioinformatics analysis of RNA-seq data

Reads were processed as described previously for the genome libraries and evaluation and filtration of the rRNAs were performed using SortmeRNA. The filtered data were mapped into the *L. sulphureus* ATCC 52600 reference genome sequenced in this work using the Tophat2 algorithm [97]. Differential gene expression analysis was based on counting data and performed with the Bioconductor DESeq2 package [98] using the R platform, by paired comparisons against the control condition. Transcripts showing differential expression (log2-fold change ≥ 1 and ≤ − 1) relative to the non-induced condition (T0) were determined by applying *p* ≤ 0.05 as the threshold.

### Carbohydrate metabolism and glucose consumption

Mycelia discs were excised from the border of the colony growing on potato dextrose agar plates and transferred to the center of minimal medium agar plates [99] supplemented with 1% (w/v) of the following substrates: glucose, arabinose, galacturonic acid, xylose, lactose, cellobiose, galactose, xylan from beechwood, pectin from citrus, and galactomannan from carob. Cultivation was performed in six replicates for 7 days at 30 °C, and growth rates were estimated from the daily measurement of the colony area using the software ImageJ 1.52a [100]. For cultivation in liquid medium, 15 mycelial discs were transferred into 250-mL Erlenmeyer flasks containing 100 mL of liquid minimal medium pH 5.5 supplemented with 1% (w/v) glucose for 7 days under static conditions at 30 °C. Cultivation was performed in triplicate and samples were taken at 6, 12, 24, 48, 72, 96, and 120 h of cultivation. Residual glucose was measured by high-performance liquid chromatography (HPLC), as described below.

### Cultivation conditions for proteomic analysis

#### Pre-inoculum

*L. sulphureus* ATCC 52600 was grown on potato dextrose agar plates pH 5.5 at 25 °C. After 7 days of cultivation, 15 mycelium discs (8 mm diameter) were excised from the colony border and transferred to 250-mL Erlenmeyer flasks containing 50 mL of liquid medium composed of 0.5 g/L NH_4_CH_3_CO_2_, 0.5 g/L NaNO_3_, 0.5 g/L MgSO_4_, 0.2 g/L Na_2_HPO_4_, 0.8 g/L KH_2_PO_4_, 4.0 g/L yeast extract, and 10.0 g/L glucose. The pre-inoculum was incubated for 21 days under static conditions at 30 °C.

#### Cultivation

Pre-grown mycelia were removed by filtration, washed with distilled water, transferred to a 50-mL conical tube, and manually macerated with 2 g of glass beads. The macerated mycelia were then transferred to 250-mL Erlenmeyer flasks containing 50 mL minimal medium pH 5.5 supplemented with 1% (w/v) steam-exploded sugarcane bagasse (SCB), steam-exploded sugarcane straw (SCS), steam-exploded *Eucalyptus* residue (*Eucalyptus grandis*), Avicel® PH-101 (Sigma), and glucose. Cultivation was performed in triplicate under static conditions for 7 days at 30 °C.

### Mass spectrometry and data analysis

Cultivation supernatants (secretomes) were filtered with Miracloth (Millipore), centrifuged (13,000* g*, 20 min, 4 °C), and concentrated using 10-kDa cut-off Amicon Centrifugal Filter Units (Millipore). Protein concentration was measured with the Pierce BCA Protein Assay kit (Thermo Scientific) using BSA as standard. Secretomes (20 µg) partially resolved on 12% SDS-PAGE [101] were excised, reduced, and digested with 20 mg/ml trypsin (Promega) [102]. After extraction, samples were dried under vacuum and peptide mixtures were analyzed in LTQ Velos Orbitrap-activated, as described elsewhere [103].

Spectra data were annotated based on the *L. sulphureus* ATCC 52600 genome. The adjusted conditions to validate protein identification were protein probability thresholds higher than 99% and at least 2 different peptides identifying a protein, each with 95% certainty. Once the parameters were defined, a 0.0% false discovery rate (FDR) was generated and spectrum count data were analyzed in a semi-quantitative method. Spectra counts are equivalent to the total number of standard spectra assigned to each protein and are commonly used to determine relative abundance [104]. As the spectra counting methodology was used for analysis, FDR was designated as one of the parameters to determine the reliability of the experimental data. FDR was defined as the expected correspondent percentage of each peptide spectrum [105]. Initially, a score was assigned to each peptide (primary analysis) performed with Mascot Distiller software. Subsequently, Mascot data were analyzed by Scaffold 4 Proteomic software attributing the number of spectra to the abundance and FDR to the reliability of the results. By using average spectra outputs from Scaffold 4, differentially secreted proteins were identified according to their spectra counting and quantitative values were applied to normalize the counts. The statistical analysis of the spectra was performed by the t-test (*p* ≤ 0.05) and fold change by category, using data from cultivation with glucose as standard.

GO terms were analyzed and identified in the topGO platform (https://bioconductor.org/packages/release/bioc/html/topGO.html) using the following tools: basic local alignment search (BLAST) (https://blast.ncbi.nlm.nih.gov/Blast.cgi), PFAM (https://pfam.xfam.org/), and MEROPS (https://merops.sanger.ac.uk/). Parameters used to run BLASTp were: E-value ≤ 40, identity ≥ 40%, and consultation coverage ≥ 80%. Classification of CAZymes and carbohydrate-binding modules (CBM) was performed on dbCAN v.8 (www.csbl.bmb.uga.edu/dbCAN). The presence of at least three representative members was established to define a classification group. Prediction of signal peptide (SP) and non-classical protein secretion were verified using SignalP 4.1 (www.cbs.dtu.dk/services/SignalP) and SecretomeP 2.0 (www.cbs.dtu.dk/services/SecretomeP/), respectively.

### Activity on different substrates

Enzymatic assays were performed using 50 μl of the following substrates: 5 mM 4-nitrophenyl β-D-cellobioside, 4-nitrophenyl β-D-xylopyranoside and 4-nitrophenyl β-D-glucopyranoside, and 0.5% (w/v) polygalacturonic acid, starch, CMC, xylan from beechwood (Sigma), wheat arabinoxylan, β-glucan and galactomannan (Megazyme). Assays were performed using 1 μg protein of the concentrated secretomes in 50 mM ammonium acetate buffer pH 5.5 for 4 h at 50 °C. Assays with the synthetic substrates were stopped with 100 μl of 1 M sodium bicarbonate and the released 4-nitrophenolate was measured at 405 nm. Reactions with polymeric substrates were stopped with 100 μl of 3,5-dinitrosalicylic acid (DNS) and the released reducing sugars were measured at 540 nm [106]. All assays were performed in triplicate. One enzyme unit (1 U; μmol/min) corresponds to the amount of enzyme that catalyzes the conversion of one micromole of substrate per minute under the assay conditions.

### Enzymatic saccharification

Grass (sugarcane straw) and pine softwood (*Pinus* sp) lignocellulose were milled (1.0 cm length × 1.0 mm thickness), followed by hydrothermal pretreatment and composition characterization [107, 108]. Saccharification reactions were performed in 1 mL working volume with substrate load at 2% (w/w) solids in 50 mM sodium acetate buffer pH 5.0 at 50 °C up to 48 h in a Thermomixer under 1000 rpm agitation. The *L. sulphureus* secretome produced on SCB (as described above) was evaluated by replacing 15% of protein load from commercial enzymatic cocktails. FPAse activity was previously assayed in the enzymatic cocktail [109, 110] and the total protein load (equivalent to 15 FPU) was the combination 5:1 (w/w) Celluclast®:glucosidase from *Aspergillus niger* (Merck) per gram of dry substrate (Celluclast® at 150 mg protein/mL corresponding to 115 FPU/mL). Assays were performed in four replicates and the released sugars were measured by HPLC, as described below. Biomass conversion was calculated using glucan/xylan content in the biomass (pretreated/native) and the anhydrous correction factors of 1.13 for xylose and 1.1 for glucose [108, 111, 112].

### HPLC analysis

Glucose, xylose, and cellobiose were quantified in a liquid chromatography system (Waters 515 Pump, Water 717 plus Injector/Sampler) coupled to an Aminex HPX87H (300 × 7.8 mm) column and equipped with a refractive index (RI) detector (Waters 410). Detector and column temperatures were set, respectively, to 40 and 45 °C; 50 mM H_2_SO_4_ was used as a mobile phase at 0.6 ml/min flow rate; and 20 μl injection volume.

## Supplementary Information

**Additional file 1**: **Figure S1**. Overview of *L. sulphureus* ATCC 52600 multi-omics analysis. Distribution of CAZymes and redox Non-CAZymes presented in the (A) genome and (B) transcriptome. (C) Categorization of all protein identified on the secretomes. **Figure S2**. Analysis of *L. sulphureus* ATCC 52600 growth on different carbohydrates. (A) Growth for 7 days on agar plates supplemented with different carbohydrates. (B) Growth rate estimated by colony area measurement. Values expressed relative to glucose. (C) Relative glucose consumption measured using HPLC during growth in liquid medium. Growth rates were analyzed using two-way ANOVA with Tukey’s test, indicated as follows: *p<0.05, **p<0.01, ***p<0.001. **Figure S3**. Enzymatic activity profile of *L. sulphureus* ATCC 52600 secretomes. Enzymatic assays containing the different secretomes (Avicel; SCB: sugarcane bagasse; Eucalyptus: Eucalyptus grandis residue, SCS: sugarcane straw and glucose) were carried out in 50 mM sodium acetate buffer pH 5.5 for 240 min at 50 °C. Activities were analyzed assuming the secretome produced on SCB as control by two‑way ANOVA with Tukey’s test (95% confidence interval), indicated as follows: *p<0.05, **p<0.01, ***p<0.001. Reducing sugars were measured using the DNS method. **Figure S4**. Multiple AA14 sequence alignment. The alignment was generated by Clustal using two characterized lytic polysaccharide monooxygenases (LPMOs) from *Pycnoporus coccineus* CIRM-BRFM 310 (PcAA14A and PcAA14B) and two putative AA14 found in the *L. sulphureus* ATCC 52600 genome. Red boxes highlight the conserved amino acid residues constituting the histidine brace, a hallmark of LPMOs. **Figure S5**. Multiple alignment of GH7 amino acid sequences. (A) Alignment was performed by Clustal using basidiomycete cellobiohydrolases with identity higher than 65% (GenBank: KIY52887, VDC00014, and OBZ74435). (B) Phylogenetic tree of GH7 amino acid sequences, which includes 14 characterized cellobiohydrolases according to UniProt, which are from *Aspergillus aculeatus*, *Aspergillus niger*, *Aspergillus terreus*, *Aspergillus fischerianus*, *Aspergillus fumigatus*, *Aspergillus nidulans*, *Penicillium funiculosum*, and *Phanerochaete chrysosporium*.

**Additional file 2**: **Table S1**. Main genes involved in brown-rot wood decay identified in *L. sulphureus* ATCC 52600 genome. **Table S2**. Differential expression of CAZymes in the transcriptome of L. sulphureus ATCC 52600. **Table S3**. Proteins identified with statistical significance in the exoproteomes of L. sulphureus ATCC 52600 cultivated on different substrates.

## Data Availability

The datasets generated for this study can be found in the Gene Expression Omnibus with the GEO accession number GSE151004.
