# Supplementary material for: Multi-omics analysis provides insights into lignocellulosic biomass degradation by Laetiporus sulphureus ATCC 52600
Source: Biotechnol Biofuels. 2021 Apr 17;14:96. doi: 10.1186/s13068-021-01945-7 (PMC8052766; doi:10.1186/s13068-021-01945-7)
Supplement: Supplementary file 1 — Additional file 1: Figure S1. Overview of L. sulphureus ATCC 52600 multi-omics analysis. Distribution of CAZymes and redox Non-CAZymes presented in the (A) genome and (B) transcriptome. (C) Categorization of all protein identified on the secretomes. Figure S2. Analysis of L. sulphureus ATCC 52600 growth on different carbohydrates. (A) Growth for 7 days on agar plates supplemented with different carbohydrates. (B) Growth rate estimated by colony area measurement. Values expressed relative to glucose. (C) Relative glucose consumption measured using HPLC during growth in liquid medium. Growth rates were analyzed using two-way ANOVA with Tukey’s test, indicated as follows: *p<0.05, **p<0.01, ***p<0.001. Figure S3. Enzymatic activity profile of L. sulphureus ATCC 52600 secretomes. Enzymatic assays containing the different secretomes (Avicel; SCB: sugarcane bagasse; Eucalyptus: Eucalyptus grandis residue, SCS: sugarcane straw and glucose) were carried out in 50 mM sodium acetate buffer pH 5.5 for 240 min at 50 °C. Activities were analyzed assuming the secretome produced on SCB as control by two‑way ANOVA with Tukey’s test (95% confidence interval), indicated as follows: *p<0.05, **p<0.01, ***p<0.001. Reducing sugars were measured using the DNS method. Figure S4. Multiple AA14 sequence alignment. The alignment was generated by Clustal using two characterized lytic polysaccharide monooxygenases (LPMOs) from Pycnoporus coccineus CIRM-BRFM 310 (PcAA14A and PcAA14B) and two putative AA14 found in the L. sulphureus ATCC 52600 genome. Red boxes highlight the conserved amino acid residues constituting the histidine brace, a hallmark of LPMOs. Figure S5. Multiple alignment of GH7 amino acid sequences. (A) Alignment was performed by Clustal using basidiomycete cellobiohydrolases with identity higher than 65% (GenBank: KIY52887, VDC00014, and OBZ74435). (B) Phylogenetic tree of GH7 amino acid sequences, which includes 14 characterized cellobiohydrolases according to UniProt, which a [file 13068_2021_1945_MOESM1_ESM.docx]

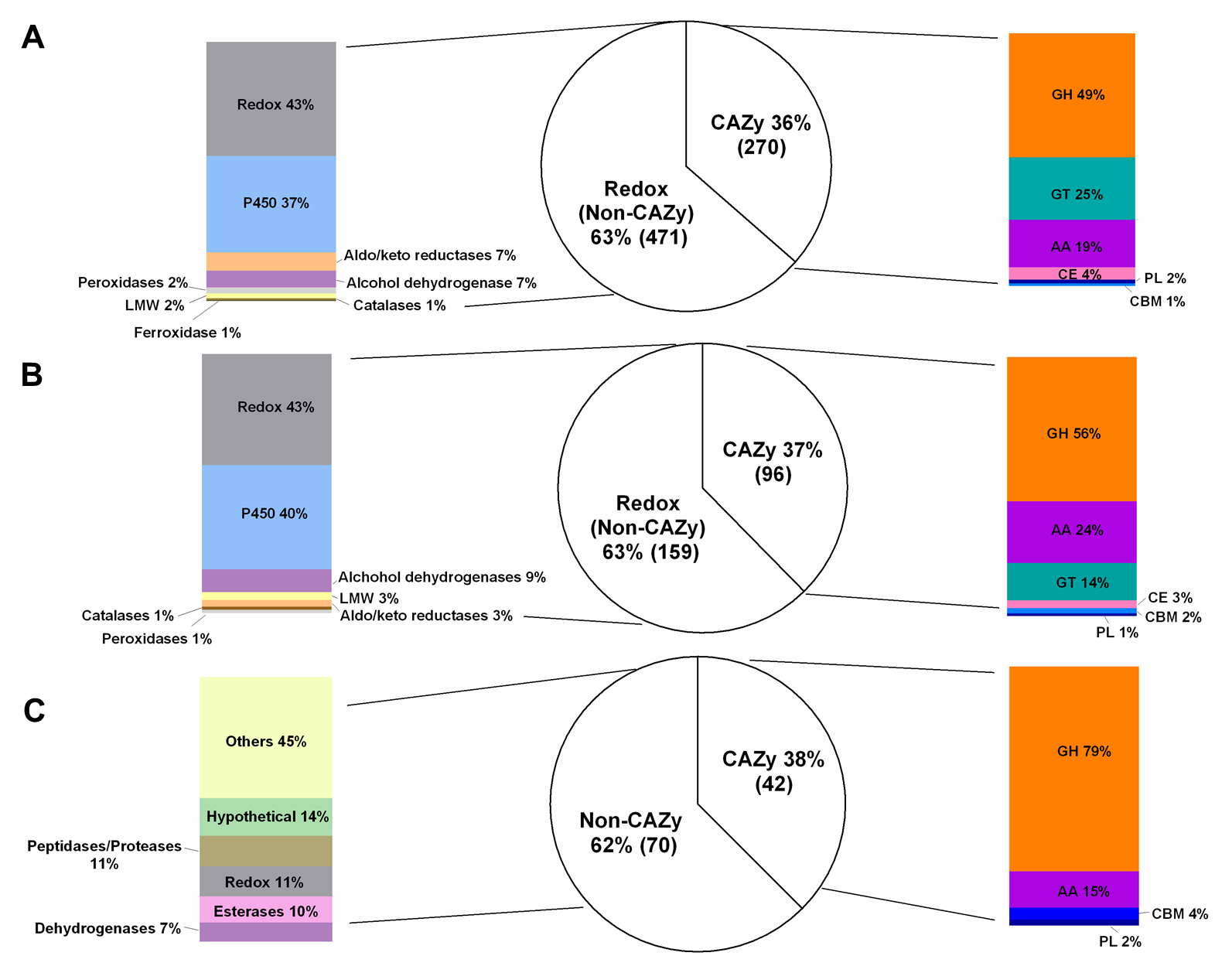


**Figure S1. Overview of *L. sulphureus* ATCC 52600 multi-omics analysis.** Distribution of CAZymes and redox Non-CAZymes presented in the (A) genome and (B) transcriptome. (C) Categorization of all protein identified on the secretomes.

**
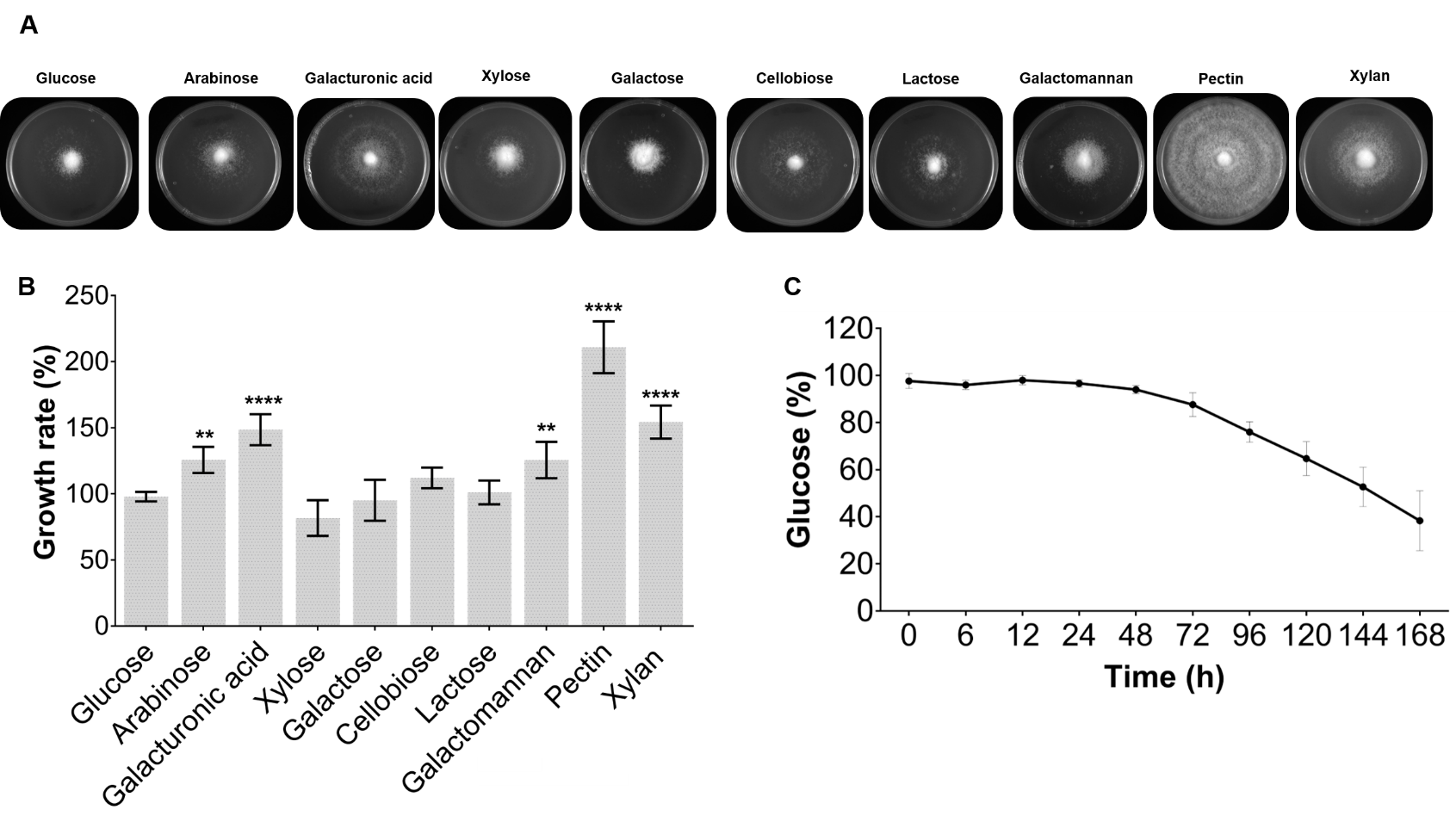
Figure S2. Analysis of *L. sulphureus* ATCC 52600 growth on different carbohydrates.** (A) Growth for 7 days on agar plates supplemented with different carbohydrates. (B) Growth rate estimated by colony area measurement. Values expressed relative to glucose. (C) Relative glucose consumption measured using HPLC during growth in liquid medium. Growth rates were analyzed using two-way ANOVA with Tukey’s test, indicated as follows: **p*<0.05, ***p*<0.01, ****p*<0.001.

**
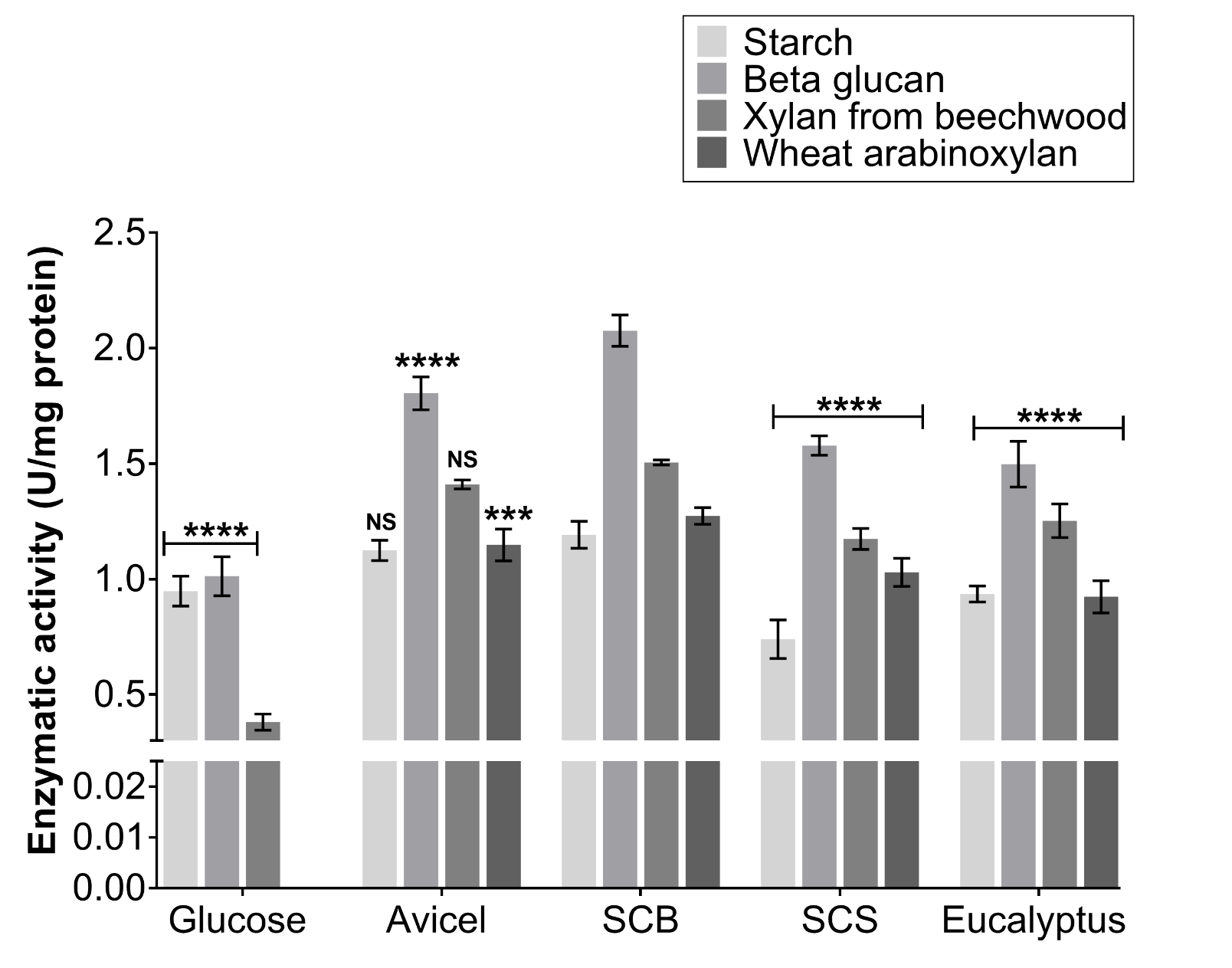
**

**Figure S3. Enzymatic activity profile of *L. sulphureus* ATCC 52600 secretomes.** Enzymatic assays containing the different secretomes (Avicel; SCB: sugarcane bagasse; Eucalyptus: *Eucalyptus grandis* residue, SCS: sugarcane straw and glucose) were carried out in 50 mM sodium acetate buffer pH 5.5 for 240 min at 50 °C. Activities were analyzed assuming the secretome produced on SCB as control by two‑way ANOVA with Tukey’s test (95% confidence interval), indicated as follows: **p*<0.05, ***p*<0.01, ****p*<0.001. Reducing sugars were measured using the DNS method.


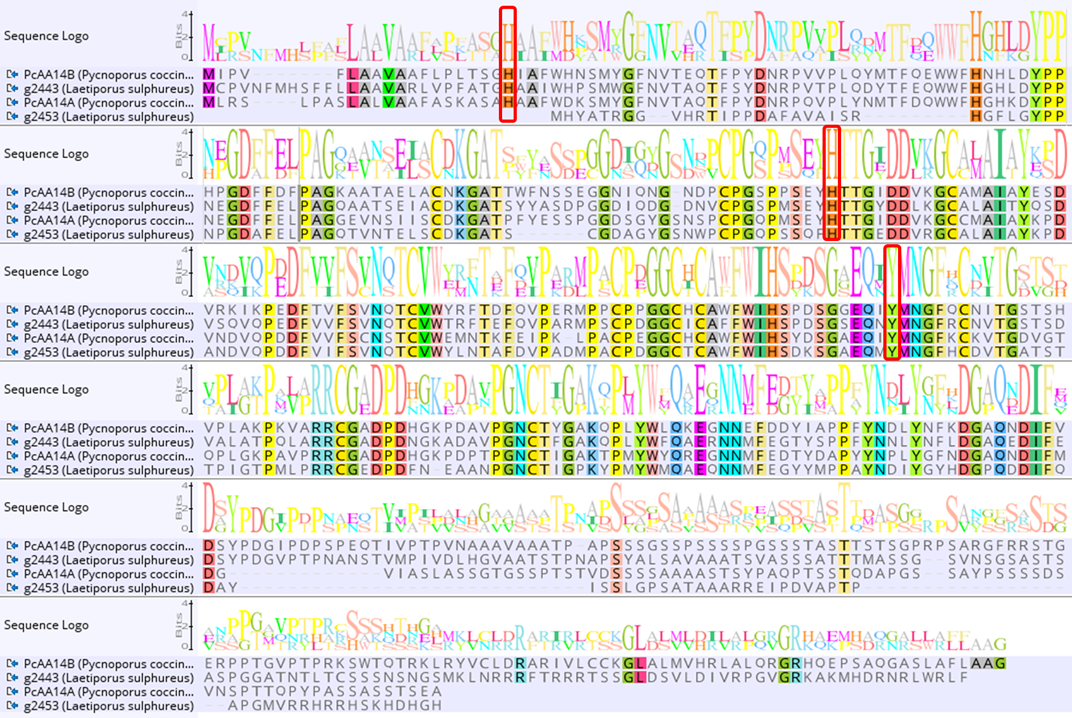


**Figure S4. Multiple AA14 sequence alignment.** The alignment was generated by Clustal using two characterized lytic polysaccharide monooxygenases (LPMOs) from *Pycnoporus coccineus* CIRM-BRFM 310 (PcAA14A and PcAA14B) and two putative AA14 found in the *L. sulphureus* ATCC 52600 genome. Red boxes highlight the conserved amino acid residues constituting the histidine brace, a hallmark of LPMOs.


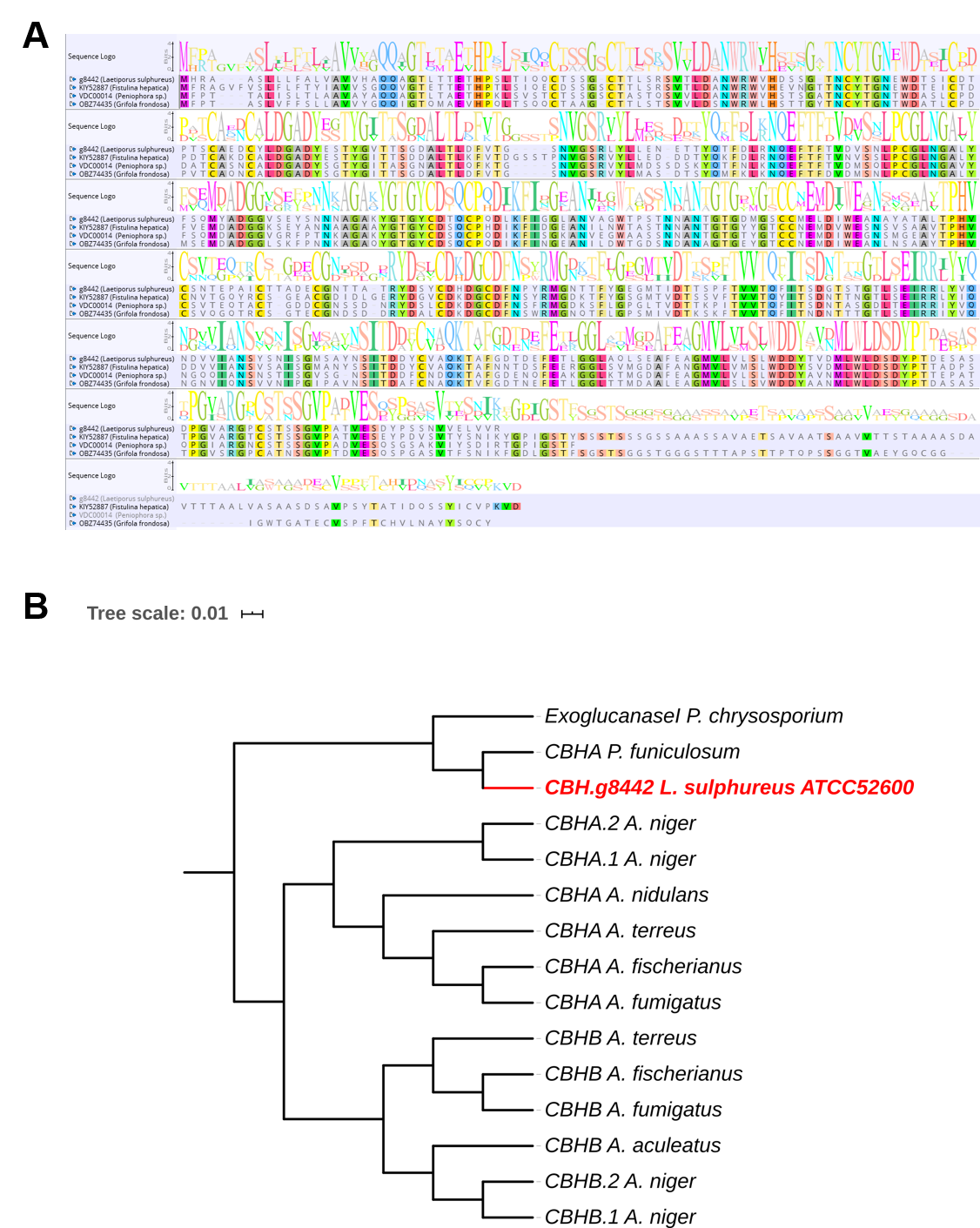


**Figure S5. Multiple alignment of GH7 amino acid sequences.** (A) Alignment was performed by Clustal using basidiomycete cellobiohydrolases with identity higher than 65% (GenBank: KIY52887, VDC00014, and OBZ74435). (B) Phylogenetic tree of GH7 amino acid sequences, which includes 14 characterized cellobiohydrolases according to UniProt, which are from *Aspergillus aculeatus*, *Aspergillus niger*, *Aspergillus terreus*, *Aspergillus fischerianus*, *Aspergillus fumigatus*, *Aspergillus nidulans, Penicillium funiculosum,* and *Phanerochaete chrysosporium*.
